# Extracellular superoxide production by *Porites* species provides insight into controls on coral physiology

**DOI:** 10.1093/pnasnexus/pgag075

**Published:** 2026-03-19

**Authors:** Kalina C Grabb, Santiago Herrera, Loretta M Roberson, Mayra Sánchez-García, Heather N Page, Colleen M Hansel

**Affiliations:** MIT-WHOI Joint Program in Oceanography/Applied Ocean Science and Engineering, Cambridge and Woods Hole, MA 02139, USA; Marine Chemistry and Geochemistry, Woods Hole Oceanographic Institution,Woods Hole, MA 02543, USA; Marine Chemistry and Geochemistry, Woods Hole Oceanographic Institution,Woods Hole, MA 02543, USA; Biological Sciences, Lehigh University, Bethlehem, PA 18015, USA; Marine Biological Laboratory, Bell Center, Woods Hole, MA 02543, USA; Marine Biological Laboratory, Bell Center, Woods Hole, MA 02543, USA; Sea Education Association, Falmouth, MA 02540, USA; Marine Chemistry and Geochemistry, Woods Hole Oceanographic Institution,Woods Hole, MA 02543, USA

**Keywords:** reactive oxygen species, coral, *Porites* species, NADPH oxidase, extracellular superoxide

## Abstract

Reactive oxygen species (ROS), including superoxide, are central molecules in eukaryotic growth, function, and immunity. Some coral species, such as *Porites* sp., have been associated with high extracellular superoxide concentrations, yet we lack a clear understanding of the role of controls on superoxide production in corals. Here, we combine extracellular superoxide concentration and decay rate measurements with bioinformatics to better constrain the controls and mechanisms underlying ROS formation by *Porites* species. Consistent with previous studies, we find that extracellular superoxide concentrations are significantly higher for *Porites* species compared with other coral species. We further find that superoxide decay rates are not significantly different across species, indicating that changes in production rather than decay control steady-state concentrations. Extracellular superoxide is produced by *Porites astreoides* across life stages from larval to newly settled polyps to adult colonies. Our bioinformatic analysis reveals that *Porites lobata* has genes that encode for two types of NADPH oxidase (NOX), enzymes that produce exclusively extracellular superoxide. In fact, we find widespread presence of NOX genes within genomes across scleractinian species despite species-specific variation in superoxide production. Together, these findings indicate that corals regulate extracellular superoxide levels and point to an important role for extracellular superoxide in coral physiology, such as cell signaling, cell differentiation, and growth regulation. These findings add to a growing appreciation for the beneficial role of ROS in marine organisms and provide a foundation to investigate the role of ROS in coral physiology that may provide insight into coral health and subsequent approaches for coral restoration and preservation.

Significance statement Reactive oxygen species (ROS) are a suite of chemicals that are both beneficial and detrimental to organisms. Previous studies found that the levels of the ROS superoxide outside coral cells substantially vary as a function of coral species. Here, we show that the highest superoxide-producing species, *Porites* species, produces extracellular superoxide throughout its life cycle. The known ROS-producing enzyme NADPH oxidase (NOX) is widespread across corals, even those associated with low superoxide concentrations. Thus, this study indicates that corals control the production of superoxide at their surfaces, likely in response to environmental conditions and physiological needs. This research lays the groundwork for future studies to directly examine NOX regulation and its role in coral health and disease resistance.

## Introduction

Reactive oxygen species (ROS) are short-lived oxygen-bearing molecules (1) that serve essential physiological roles within organisms, including animals, plants, and microbes (see (2) and references therein). The ROS superoxide ($O_{2}^{∙-}$) and hydrogen peroxide (H_2_O_2_) are produced both intracellularly (inside the cell) and extracellularly (outside the cell) by a broad range of marine macro- and micro-organisms, such as algae, bacteria, phytoplankton, and seaweed (3–7). At elevated concentrations, intracellular ROS can lead to oxidative stress, causing damage to lipids, proteins, and DNA (8, 9). However, lower concentrations of ROS both within and outside the cell are involved in various physiological processes and biological interactions (10), including growth regulation (11), cell signaling (12), micronutrient acquisition (13), pathogen defense (14), and heat stress regulation (15). Within biological systems, hydrogen peroxide actively or passively transports across cell membranes, while superoxide cannot diffuse across intact biological membranes (16, 17). Thus, intracellular and extracellular superoxide production is spatially separated, whereby extracellular superoxide is a consequence of reactions mediated by soluble metabolites and/or outer membrane or transmembrane enzymes, rather than intracellular processes such as symbiont activity (18, 19).

Despite the misconception that ROS are solely stress molecules within corals, there is evidence that ROS production by corals also plays beneficial roles (20–22). For instance, light- and temperature-induced stress has been implicated in the overproduction of intracellular ROS by the endosymbiotic algae Symbiodiniaceae, leading to oxidative stress, bleaching, and possibly apoptosis (cell death) of the coral host (8). In contrast, recent research found that intracellular ROS production did not cause coral symbiont expulsion (23), heat stress did not necessarily spur intracellular ROS production (24), and ROS may play a role in molecular signaling between coral host and endosymbionts (25). Coral host extracellular ROS production has also been associated with several beneficial physiological processes (e.g. food acquisition, increased thermotolerance, oxidative stress reduction, and wound repair) and pathogen (e.g. *Vibrio shiloi*) and disease (e.g. white band) resistance (20, 21, 26, 27).

The only two previous studies that measured extracellular superoxide associated with scleractinian corals in situ within natural reefs found species-specific concentrations at colony surfaces in reefs within the Pacific (28) and Caribbean (29), suggesting these trends are independent of geographic location. More specifically, steady-state extracellular superoxide concentrations associated with some coral species (i.e. *Pocillopora damicornis*, *Porites astreoides*, *Porites compressa*, *Porites lobata*, and *Porites porites*, ∼25 to ∼173 nM) were higher than those found for other marine organisms (4, 10, 30). In contrast, some corals had extracellular superoxide levels that were low (*Fungia scutaria*, <5 nM) or similar to background seawater (BGSW; *Orbicella faveolata*, *Montastraea cavernosa*, and *Montipora capitata*, ≲0 nM) (28, 29). In these studies, extracellular superoxide was associated with healthy, pigmented corals (28, 29) and was independent of light conditions, algal abundance, and bleaching status, indicating that extracellular ROS was not produced via a stress response (17, 20, 27, 31). Further, removal of the coral surface mucus layer and associated microbes did not change measured extracellular superoxide levels (31). In fact, a connection between the composition or abundance of microbes on the coral surface and coral-associated superoxide was not observed, thereby pointing to the coral host as the primary source of superoxide.

The underpinning production mechanisms and physiological basis for extracellular superoxide production by corals remain poorly understood. Enzymes belonging to the NADPH oxidase (NOX) family generate extracellular ROS as their sole function, and there is no evidence of multicellular life without NOX enzymes (32). NOX-dependent extracellular superoxide production was bioinformatically predicted and empirically observed in a wide range of eukaryotes, including phytoplankton (27, 33), seaweed (7, 15), algae (34), the sea anemone *Nematostella vectensis* (35–37) and, more recently, deep-sea corals and sponges (38). Several NOX homologs have evolved across organisms (NOX1–5 and DUOX) with specific regulatory subunits (39). There has been limited research on NOX enzymes or genes within coral genomes, yet two previous studies observed expression of a NOX3 homolog within *Acropora cervicornis* and two ancestral NOX2 genes within *N. vectensis* (21, 40). Further, a recent study identified both NOX and DUOX genes within deep-sea corals and sponges (38). Given the prevalence of NOX genes across eukaryotic life and observations of extracellular superoxide production by some coral species, NOX-derived extracellular superoxide production by corals is likely a widespread process. However, the presence of NOX across scleractinian coral species has yet to be investigated.

Accordingly, the goal of this study is to better constrain the controls on extracellular superoxide concentrations associated with scleractinian corals, with a focus on *Porites* species. It is of interest to better understand the physiological controls of *Porites* species since various *Porites* are consistently associated with high superoxide levels (28, 29), have increased resistance to diseases such as the stony coral tissue loss disease (41), and have increased in relative abundance despite the overall decline in coral cover (42). For the first time, we couple in situ steady-state concentration measurements with decay rates to quantify production rates associated with *Porites* and other species. We also quantify extracellular superoxide associated with *Porites* across life stages. Lastly, we use bioinformatic analysis to confirm the genetic potential for extracellular superoxide production by *Porites* species and other scleractinian corals. Based on our findings, we suggest that corals tightly control extracellular superoxide production via NOX enzymes throughout their life cycle and recommend that future studies specifically target the link between the regulation of NOX activity and extracellular superoxide across species and in relation to other physiological processes. Considering the accepted role of extracellular superoxide in organismal health more broadly, further investigation into these regulatory controls may provide critical insight into processes that could improve coral immunity and subsequent coral reef health.

## Results

### In situ extracellular superoxide associated with corals

#### In situ extracellular superoxide measurements

**Figure 1:**
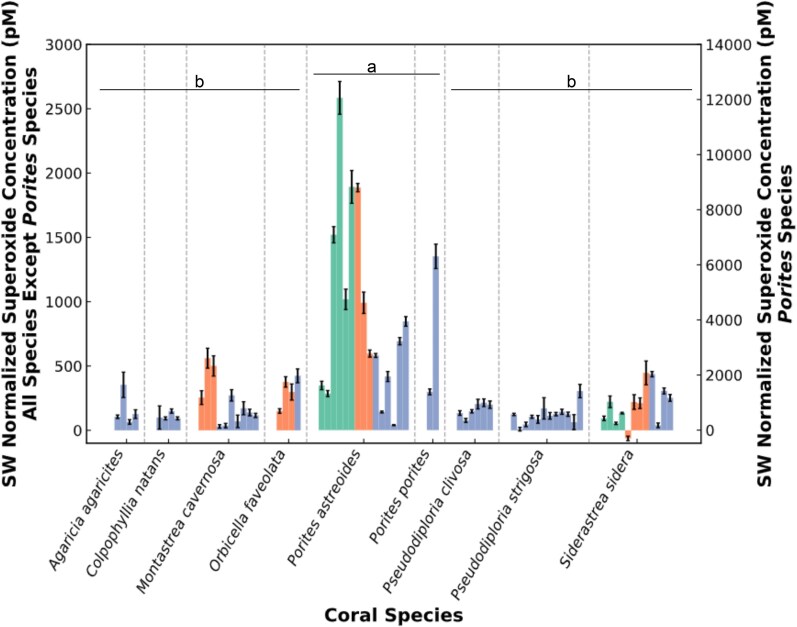
SW normalized in situ superoxide concentrations (pM, *y*-axis) associated with coral individuals across species (*x*-axis, average per individual with error bars representing SD). Colors represent sample sites: Looe Key Reef A (green), Looe Key Reef B (orange), and Dry Tortugas (blue). Vertical dashed lines separate species with groups “a” (right y-axis) and “b” (left y-axis) representing statistically different steady-state superoxide measurements between species (p-tukey values between 0.001–0.004 for *P. astreoides* and 0.131–0.326 for *P. porites*).

Seawater (SW) normalized in situ extracellular superoxide concentrations associated with coral colonies showed greater variability among species than reef sites. Concentrations ranged from a species average of 109 pM (*Colpophyllia natans*) to 4,303 pM (*P. astreoides*) above BGSW values, which is nearly 17 times higher than BGSW concentrations (Fig. 1, Tables S1 and S2). For species sampled at multiple reef sites in Florida, USA (Looe Key Reef A, LKA; Looe Key Reef B, LKB; and Dry Tortugas, DT; see Materials and methods), there was no statistical difference in extracellular superoxide concentrations between sites (ANOVA single factor, *P*-value: *O. faveolata* = 0.378, *M. cavernosa* = 0.902, *P. astreoides* = 0.128, and *Siderastrea siderea* = 0.521). Rather, there was a statistically significant difference in extracellular superoxide concentrations between species (ANOVA single factor, *P* < 0.001). The high extracellular superoxide measurements associated with *P. astreoides* (average 4,303 ± 3,338 pM, 16.7 ± 20.1 times higher than BGSW; mean ± SD) and *P. porites* (average 3,848 ± 2,460 pM, 13.1 ± 4.4 times higher than BGSW; mean ± SD) were the main drivers in this species-specific variation (post hoc Tukey test *P*-values compared with other species, default effect size of hedges: *P. astreoides* = 0.001–0.004, *P. porites* = 0.131–0.326). All other species did not differ from each other significantly (Tukey, *P* = 0.9) and had extracellular superoxide concentrations on average only 332 ± 899 pM higher than BGSW (equivalent to 1.7 times higher than BGSW; Fig. 1).

#### In situ extracellular superoxide decay rates

**Figure 2:**
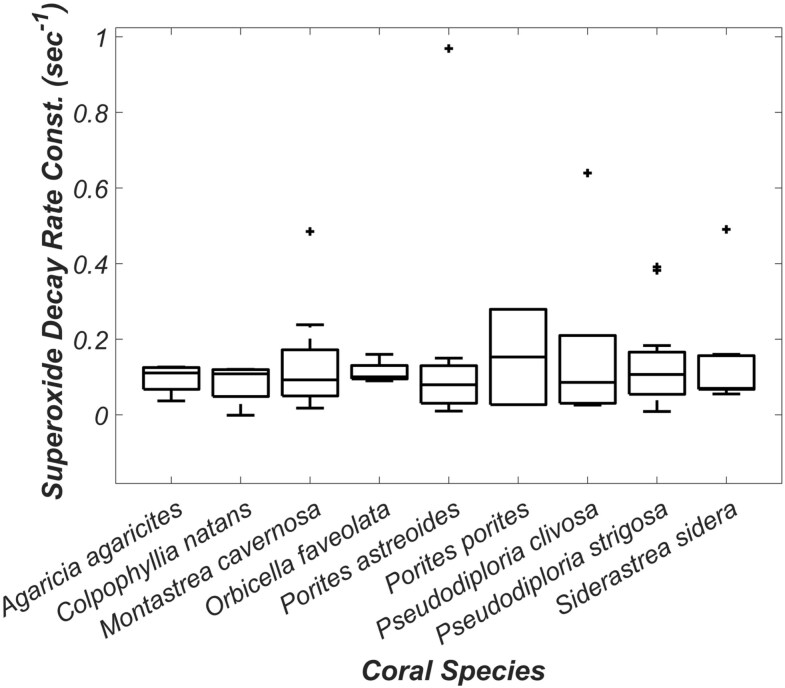
Superoxide decay rate constants (s^−1^, *y*-axis) for coral individuals across species (*x*-axis). The box and whisker plot indicates the middle 50 percentile (box), the median (horizontal line), the middle 90 percentile (vertical lines), and the outliers (“+”). *Porites porites* only had two coral individuals and, therefore, no vertical lines. There was no statistical difference in decay rate constants per species (ANOVA single factor, *P* = 0.997).

In situ extracellular superoxide decay rate constants averaged 0.140 ± 0.161 s^−1^ across all individuals (Fig. 2, Table S1). There were no statistical differences across decay rate constants in relation to coral species (ANOVA single factor, *P* = 0.997) or reef sites within the same species (ANOVA single factor, *P* = 0.279–0.768). Decay rate constants from on- and off-reef water (average 0.083 ± 0.061 and 0.034 ± 0.058 s^−1^, respectively) did not differ significantly from decay rate constants associated with individual coral surfaces (ANOVA single factor, *P* = 0.744).

### Extracellular superoxide associated with aquaria-hosted *P. astreoides* during development

#### Swimming larvae

**Figure 3:**
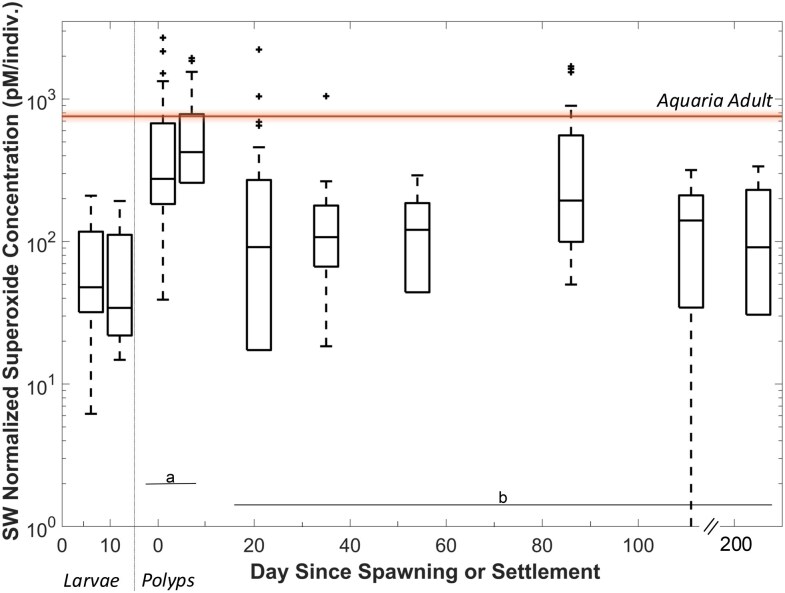
SW normalized superoxide concentration (pM/individual) for swimming larvae (on the left) and settled polyps (on the right). Data are displayed per day of sampling, with the *x*-axis indicating the number of days since spawning for swimming larvae (left) and the days since settlement for settled polyps (right). The boxes represent the middle 50 percentiles, with the horizontal line indicating the median. The middle 90 percentiles are represented by the vertical lines. The outliers are indicated by “+.” The “a” and “b” represent groups that are statistically different from one another (ANOVA, *P* = 1.3 × 10^−4^). The horizontal orange line represents the average concentration of EC-superoxide associated with adult *P. astreoides* corals hosted in aquaria (774.5 ± 404.1 pM).

Approximately 125 swimming *P. astreoides* larvae were monitored for 2 weeks and analyzed on day 8 and day 11 after spawning. Measurements were conducted using a range of larval densities (1, 5, 15, and 30 larvae per measurement). Swimming larvae exhibited steady-state extracellular superoxide concentrations per larva of 74.3 ± 60.7 and 71.8 ± 58.2 pM larva^−1^ on day 8 and day 11, respectively (Fig. 3). Extracellular superoxide production rates associated with these same larvae were 1.6 × 10^7^ and 1.4 × 10^7^ amol larva^−1^ h^−1^ on day 8 and day 11, respectively. Superoxide production by *P. astreoides* swimming larvae exhibited a density-dependent relationship, with production rates decreasing from an average of 1.5 × 10^7^ (density of one larva) to 3.0 × 10^6^ amol larva^−1^ h^−1^ (density of 30 larvae; Fig. S4).

#### Settled polyps

Within 24 h of placing 44 *P. astreoides* swimming larvae onto tiles to settle, all the larvae were settled in a fixed position, distributed amongst four tiles (Fig. S2), and had metamorphosed into a polyp, indicating a 100% settling rate. At 91 days, 43 out of the original 44 polyps remained alive, and 33% had divided into two to four polyps. Polyps remained visually healthy until the end of the experiment.

*Porites astreoides* newly settled polyps (i.e. within the first week of settlement) had significantly higher extracellular superoxide concentrations compared with polyps after 3 weeks of settling (ANOVA, *P* < 0.001). For the newly settled polyps, the average extracellular superoxide concentration for all individuals with one polyp was 610.0 ± 13.0 and 776.0 ± 21.0 pM on days 1 and 7, respectively (Fig. 3). Comparably, after 3 weeks of settling, the average extracellular superoxide concentrations ranged between 120.0 ± 96.0 and 266.0 ± 16 pM. Overall, the extracellular superoxide concentrations associated with each individual polyp varied between sample days, with no significant trend between individuals or the number of polyps (*R*^2^ < 0.4 on each sample day). The background superoxide concentrations associated with the tiles were measured on each sample day and were substantially lower than those associated with the coral polyps, with an average tile-associated superoxide concentration of 89.5 ± 30.5 pM with no notable trend between tiles or sample days. Given the widespread ability of microbes, including bacteria, to produce extracellular superoxide (3), microbial biofilms that develop on the surface of the tiles are the likely source of this background production.

#### Adult corals

Extracellular superoxide concentrations associated with adult *P. astreoides* hosted within aquaria were 774.5 ± 404.1 pM (Fig. 3). On average, this is lower than superoxide associated with *P. astreoides* within the field (∼4–170 nM, Fig. 1 (28, 29)) and more similar to concentrations associated with settled polyps during the first days of settlement (∼700 pM, Fig. 3).

### NOX gene analysis

**Figure 4:**
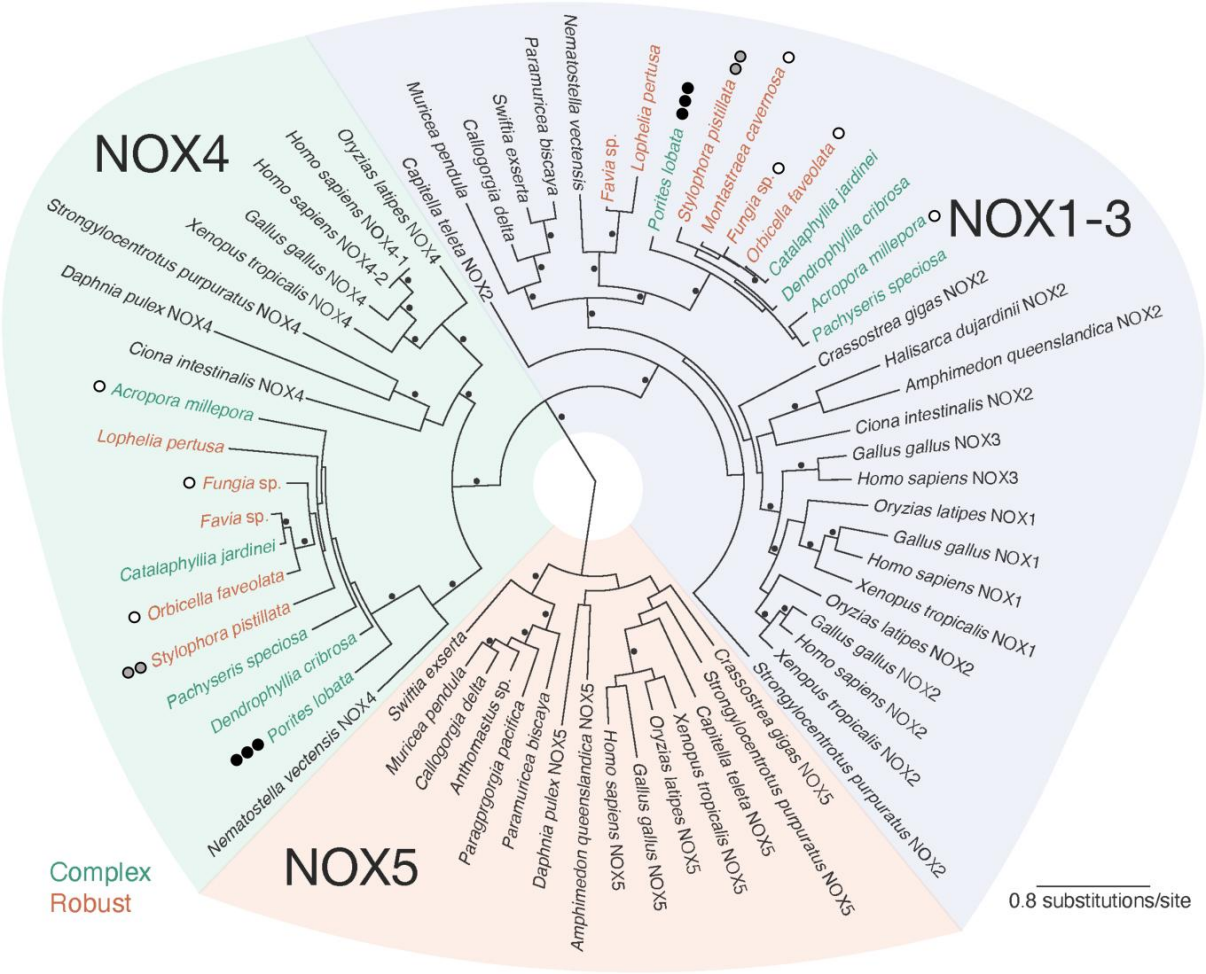
Maximum likelihood phylogenetic tree of NOX genes in corals, including reference animal genes. Scleractinian corals are indicated with green (complex clade) and orange (robust clade) color fonts. The alternating green-, blue-, and orange-shaded background areas indicate the clades of NOX gene types. Black circles in the tree indicate nodes with >90% bootstrap support. The circles adjacent to coral names indicate the relative levels of extracellular superoxide (high—three black circles, medium—two gray circles, or low—one white circle) associated with the species that have associated measurements (28, 29).

Consistent with the findings of Taenzer et al. (38), we found NOX gene types NOX2 and NOX4 in nearly all scleractinian corals queried here (Figs. 4 and S5, Table S2). The only genome that did not contain NOX4 in addition to NOX2 was that of *M. cavernosa*. No complete DUOX genes were found in any of the analyzed scleractinian corals.

#### Phylogenetic tree for coral NOX-like protein sequences

NOX2 and NOX4 scleractinian coral sequences cluster tightly and are distinct from NOX1–3 and NOX4 references from outgroup organisms (e.g. vertebrates; Fig. 4). The scleractinian coral NOX sequences form monophyletic clades (separated by >90% bootstrapping) with other anthozoan cnidarians. NOX2 and NOX4 scleractinian sequences are sister to NOX2 and NOX4 from the sea anemone *N. vectensis*, respectively, and together form a hexacoral NOX2 and NOX4 clade that is reciprocally monophyletic to the NOX2 and NOX4 from octocorals (e.g. *Swiftia exserta*), respectively. Hexacorals, including scleractinian coral, lack the NOX5 gene found in octocorals and other invertebrates and vertebrates. There were no differences in the scleractinian NOX genes consistent with the robust and complex evolutionary clades (43) (Figs. 4 and S5).

## Discussion

The goal of this study was to better constrain the controls on elevated levels of extracellular superoxide associated with *Porites* species. Similar to previous studies (28, 29), we confirm that steady-state extracellular superoxide concentrations vary across coral species with *Porites* species associated with significantly higher concentrations (Fig. 1). In contrast, we show that superoxide decay rate constants are not significantly different across species (Fig. 2). Production of extracellular superoxide spanning *P. astreoides* life cycle from larva to adult suggests that, like in other eukaryotic organisms, extracellular superoxide plays a fundamental role in *P. astreoides* physiology and immunity (Figs. 3 and S4). The ability to produce superoxide throughout their lifecycle aligns with the presence of NOX2 and NOX4 genes that we identify within *Porites* species. In alignment with previous studies documenting the presence of NOX in cnidarians (36, 39), this study also confirms the presence of NOX2 and NOX4 genes across scleractinian coral species and subsequent genetic potential for all investigated scleractinian to produce superoxide (Figs. 4 and S5). Thus, additional research is required to understand the regulatory controls on NOX within corals. We place these findings within the broader context below.

### In situ extracellular superoxide production controls species-specific variation

Consistent with previous studies on Pacific and Caribbean reefs (28, 29), here we find that extracellular superoxide concentrations associated with corals vary significantly with species. Although not all species were sampled equally due to the natural abundance distribution on the reefs, this study observed more replicates across more reef locations than previous studies (28, 29) and confirmed that all species measured within this study have extracellular superoxide concentrations that are elevated above the BGSW (e.g. *M. cavernosa*, *O. faveolata*, and *C. natans*, ∼100–300 pM) (2 , 4 , 10). Some species, such as *Porites* sp., have significantly higher extracellular superoxide concentrations (∼4,000 pM) than other coral species and marine organisms (Fig. 1) (4, 10, 30). In addition to these inter-species differences, there is also a high degree of intra-species variability (e.g. average extracellular superoxide associated with *P. astreoides* individuals ranged from ∼180 to 12,060 pM, albeit more coral individuals were sampled for *Porites* sp. compared with other species due to availability and abundance of *Porites* on the reefs). While similar species-specific extracellular superoxide trends were observed in previous studies, the range of superoxide concentrations within this study (∼100 to ∼4,000 pM on average per species) is more than an order of magnitude lower than the superoxide concentrations associated with in situ corals previously (∼4 to ∼170 nM (28, 29)). This difference in superoxide concentrations could be due to several factors, such as extrinsic factors controlling the differential expression or regulation of NOX enzymes independent of expression. For instance, differences in coral productivity and metabolism in response to reef water nutrient composition and time of year could lead to differences in extracellular superoxide production and decay. The highest extracellular superoxide concentrations associated with corals have been measured at Jardines de la Reina in Cuba (29), which is recognized as a pristine reef site and is located at lower latitudes than the Florida Keys reefs within this study.

The steady-state superoxide concentrations measured here are a result of opposing production and decay pathways. Superoxide decay rate constants within waters at the coral surfaces (on average 0.140 ± 0.161 s^−1^ across all species) do not significantly vary as a function of coral species (ANOVA single factor, *P* = 0.997; Fig. 2). Given these uniform superoxide decay rate constants, species-specific production rates are likely responsible for the observed intra- and inter-species variability in superoxide levels at the coral surfaces. Further, the similarity in decay rate constants across coral individuals suggests that abiotic reactions rather than enzymatic processes (e.g. superoxide dismutase) are the primary decay pathway. Indeed, abiotic processes have been identified as the primary superoxide degradation pathway within marine waters (e.g. trace metals and small molecules like dissolved organic matter, DOM) (44–47). Trace metals and DOM are relatively uniform within well-mixed systems and thus we anticipate concentrations will be uniform across spatial scales larger than an individual coral colony within natural reefs. These decay rate constants (average 0.140 ± 0.161 s^−1^) are several orders of magnitude faster than those typically measured in open ocean waters (46, 48, 49). The decay rate constants measured here are more similar to coastal waters such as within the Chesapeake Bay (0.1–1.4 s^−1^), where rapid decay is attributed to abundant dissolved organic molecules such as colored DOM (CDOM), organic copper, and humic and fulvic acids (50). Similar degradation pathways may be operative within reef ecosystems, yet further research is needed to pinpoint the specific reactions involved in the observed superoxide decay.

The elevated decay rate constants measured here require us to revisit previous conclusions that corals with no measurable extracellular superoxide are not producing it. Instead, previous steady-state measurements showing negligible extracellular superoxide suggest that the corals are producing superoxide at rates similar to that of decay (28, 29). By measuring both decay rate constants and steady-state concentrations associated with corals here, calculated production rates enable comparisons between corals and other organisms. In so doing, we find that the rapid extracellular superoxide production rates by some coral species, such as *P. astreoides* (2.5 × 10^12^ amol h^−1^) and *P. porites* (1.0 × 10^12^ amol h^−1^), are orders of magnitude higher than all other coral species (averages for other species are between 3.4 × 10^10^ and 1.3 × 10^11^ amol h^−1^; Fig. S3, Table S1). Similarly, larval superoxide production rates when normalized to larvae number reveal that *P. astreoides* (∼1.5 × 10^7^ amol larva^−1^ h^−1^, Fig. S4) are one to two orders of magnitude higher than larval production rates previously measured for other coral species (e.g. *Diploria labyrinthiformis*, 6.0 × 10^5^ amol larva^−1^ h^−1^; *O. faveolata*, 8.2 × 10^5^ amol larva^−1^ h^−1^; and *C. natans*, 2.3 × 10^6^ amol larva^−1^ h^−1^) (28). We thus see a similar trend between adult and larval production rates, with *Porites* species consistently having significantly higher rates (28, 29). We can also roughly compare the surface area (calculated via photographs with a scale) normalized rates for *P. astreoides* for both adult and larvae. Using calculations of the average surface area for adult colonies measured through scaled photographs within this study (average ∼90,000 mm^2^) and coral larvae (average ∼0.5 mm^2^), we find remarkably similar surface area−normalized extracellular superoxide production rates between the two (∼1 × 10^7^ and 3 × 10^7^ amol mm^−2^ h^−1^ for adults and larvae, respectively). Similarly, the difference between phytoplankton and bacteria superoxide production rates narrows when rates are normalized to surface area (3). While a suite of biophysical, physiological, and ecological reasons could be proposed to explain these relationships, we hesitate (over)interpretating these data until further research on the biochemistry of ROS production in corals (and other marine organisms) is available and larger sample sizes can be measured.

While it is difficult to compare across organisms, particularly for adult (colonial) corals, we provide a comparison here for the coral larvae to place these rates in broader context. Comparing the data currently available for extracellular superoxide production rates by larva-normalized to cell-normalized production rates, we find that coral larvae production (∼10^5^ to 10^7^ amol larva^−1^ h^−1^, Fig. S4) is faster than the typical ranges for both heterotrophic bacteria (10^−2^ to 10^3^ amol cell^−1^ h^−1^) (3, 4, 51) and most phytoplankton (ranging from 10^−1^ to 10^4^ amol cell^−1^ h^−1^) (3, 4, 6) excluding harmful algal bloom-forming species (10^5^ amol cell^−1^ h^−1^) (10). While there are limited data for extracellular superoxide production by coral symbionts, cell-normalized rates for five species of Symbiodiniaceae ranged from 0.94 to 1,200 amol cell^−1^ h^−1^ (31). The common bacterial coral symbiont Endozoicomonas had cell-normalized superoxide production rates ranging from 0.5 to 1.0 amol cell^−1^ h^−1^. Thus, superoxide production rates by coral larvae are two to three orders of magnitude faster than even the fastest rates measured so far for their algal and bacterial symbionts. As mentioned earlier, due to the inability of superoxide to cross intact biological membranes (18, 19) and the lack of a link between bacteria in the mucus layer and coral superoxide levels, extracellular superoxide associated with corals has previously been attributed to the coral host rather than the endogenous symbionts (30). Here, the substantially higher rates of superoxide production by coral larvae compared with their symbionts suggest that coral-derived extracellular superoxide occurs across life stages.

### Superoxide may play a role in coral development

The observed extracellular superoxide production (Fig. 3) throughout the lifecycle of *P. astreoides* and other corals (also see Table S5 in Diaz et al. (28)) points to a possible role for superoxide in coral cell development. This present study shows high production rates and extracellular superoxide associated with *P. astreoides* adults in situ (production rate 2.5 × 10^12^ amol h^−1^ or 2.5 × 10^6^ pmol h^−1^, Fig. S3, Table S1), adults in aquaria (steady-state concentration on average 774.5 pM, Fig. 3), swimming larvae (production rate average 1.5 × 10^7^ amol larva^−1^ h^−1^ or 15 pmol larva^−1^ h^−1^; Figs. 3 and S4), and settled polyps (steady-state concentration ranges between 89.5 and 776.0 pM, Fig. 3). The similar extracellular superoxide production rates by coral larvae (∼10^5^ to 10^7^ amol larva^−1^ h^−1^ or 0.1 to 10 pmol larva^−1^ h^−1^; see Table S5 in Diaz et al.(28)) compared with other marine organisms (ranging from 10^−1^ to 10^8^ amol individual^−1^ h^−1^ or 10^−7^ to 10^2^ pmol individual^−1^ h^−1^ (3)) suggest that superoxide could be involved in processes that have been observed in other marine organisms during developmental stages, such as coral development and/or growth regulation, even for adult coral species (11). In fact, superoxide produced by NOX enzymes is involved in cell differentiation and growth regulation within a wide diversity of eukaryotic organisms, including fungi, algae, and plants. For example, *Saccharina japonica* and *Fucus serratus* kelp depend on ROS formation to regulate reproductive organs, embryo growth, and cell differentiation (52, 53). Corals also undergo rapid metamorphosis immediately after settling, which includes extensive morphological changes and irreversible cell differentiation to transform the swimming planula into a settled polyp (54, 55). This process is thought to be tightly controlled by the regulation and expression of numerous genes, some of which vary with coral species (54). In comparison to later time points, *P. astreoides* larvae were associated with significantly higher extracellular superoxide concentrations during the first few days of settlement (e.g. ∼700 pM superoxide within the first week compared with an average of ∼90 pM thereafter; ANOVA single factor, *P* < 0.001). These elevated concentrations associated with newly settled coral polyps were comparable in concentration to superoxide associated with adult *P. astreoides* within the aquaria (∼770 pM; Fig. 3) and suggest that ROS production may be involved with metamorphic processes. Superoxide could also serve as a cell signal, as seen before in phytoplankton and heterotrophic bacteria (30) and given the density-dependent superoxide production observed with *P. astreoides* in this study (Fig. S4) and *D. labyrinthiformis* previously (28). Together, observed extracellular superoxide associated with *P. astreoides* throughout its life stages (swimming larvae to adult colonies) suggests a fundamental role for extracellular superoxide in the physiology of *P. astreoides*.

### Two types of NOX genes are widespread across scleractinian coral

Extracellular superoxide production within eukaryotic organisms, spanning humans to plants, has been linked to the activity of NOX enzymes. In fact, a recent study also identified NOX genes within deep-sea corals and sponges and suggested the link between NOX and superoxide production (38). However, the presence of NOX genes within a diversity of scleractinian genomes has not been previously reported. Here, we reveal that all scleractinian corals investigated within this study (total of 11 species, including *P. lobata*) have both NOX2 and NOX4, except for *M. cavernosa*, where only NOX2 was identified (likely due to an incomplete genome assembly; Figs. 4 and S5, Table S2). The identification of NOX2 agrees with previous expectations that NOX2 exists within the phylum Cnidaria since it is the most conserved and ancestral NOX enzyme and is similar to “cytochrome b-245 heavy chain” (35, 36, 39). It is of no surprise that this study also identified NOX4 since the presence of NOX4-like genes has previously been suggested within other corals such as *Stylophora pistillata*, *P. damicornis*, and *Acropora millepora* (identified with the automated computational analysis within the National Center for Biotechnology Information [NCBI]). Further research is needed to measure the expression of NOX in relation to superoxide concentrations and specifically identify NOX enzymes as the source of extracellular superoxide production by scleractinians. Nevertheless, NOX enzymes are the only known enzymes to produce ROS as their sole purpose (2), and thus, extracellular superoxide production by corals is likely produced in large part by NOX.

The presence of NOX2 and NOX4 across scleractinians suggests that superoxide concentrations are not controlled by the presence of NOX alone; both NOX2 and NOX4 are present within the genomes of coral species that are high superoxide producers (i.e. *P. lobata* ∼10^12^ amol h^−1^) as well as those that produce superoxide at significantly lower rates (e.g. *O. faveolata*, *C. natans*, and likely *M. cavernosa*, ∼10^9^ to 10^10^ amol h^−1^; Fig. S3, Table S1) (2, 4, 10). While NOX2 and NOX4 are structurally similar and are likely to have similar mechanisms of regulation (56), various NOX types relate to different physiological processes and regulation, thereby leading to extracellular superoxide production under different circumstances.

Additional studies are required to investigate why *Porites* species have been repeatedly associated with significantly higher concentrations of superoxide compared with other species, despite similar genetic potential for extracellular superoxide production. To our knowledge, NOX regulation and expression controls have not been investigated within coral. In other organisms, NOX is tightly regulated by complex mechanisms involving regulatory subunits and/or calcium signaling (57). In many eukaryotes (humans, fungi, seaweeds, algae, etc.), NOX expression has been linked to various physiological processes, including cell differentiation, wound repair, and defense against pathogens and grazers (2, 14, 39, 57). Specifically, NOX2 expression in humans is an innate immune response (39), whereas tight regulation of NOX4 plays a role in many pathologies, specifically vascular pathologies in humans (58). While the specific physiological role(s) that NOX and extracellular superoxide play in coral physiology is currently unknown, this study offers intriguing evidence that calls for future research that investigate NOX activity, differential NOX expression, and/or regulation of enzymatic activity associated with superoxide production within corals. Further, other extracellular superoxide-generating processes besides NOX must also be considered, including excretion of reactive small molecules and other enzymes (e.g. glutathione reductase) (2, 59).

### Larger implications of NOX and extracellular superoxide within scleractinian coral

Overall, this research further points to a physiological role for extracellular superoxide within corals, in line with other eukaryotic organisms and in contrast to the historical view of ROS solely as harmful chemicals. In fact, this research aligns with more recent findings that suggest ROS are essential to the physiology and health of corals in processes such as prey acquisition, cell defense, and immunity (20, 22, 60). While direct evidence of the role of NOX-derived ROS in coral health is limited, a few studies have linked the presence and expression of NOX with coral thermotolerance and resistance to pathogenic white band disease in *A. millepora* (21, 61). Given the identified widespread presence of superoxide-generating NOX genes across scleractinian corals, this study points to the need for a better understanding of the role and function of NOX genes in producing superoxide within corals. If ROS play a similar role in corals as in other organisms, ROS production could serve as a key process in wound repair and could be involved in the innate immunity of coral species protecting against diseases and stress (2, 14, 57–60). Thus, this study hints at an underappreciated role of ROS in coral physiological function that requires further exploration. These findings further highlight the need to think wholistically about the role that ROS play in coral health and caution against interpreting ROS and antioxidant enzymes as indicators solely of oxidative stress.

## Materials and methods

### In situ extracellular superoxide measurements

#### In situ coral sampling

Field sampling was completed in November and December 2020 in the Florida Keys, USA, aboard the “SSV Corwith Cramer.” In situ extracellular superoxide measurements were collected from the surface of coral individuals at three reef sites: two reefs (A and B) in Looe Key (LK), FL (LKA: 24°32′44″N, 81°24′24″W and LKB: 24°32′48″N, 81°24′12″W, collected under permit FKNMS-2020-139 to H.N.P.) and one reef in the Dry Tortugas, FL (DT: 24°37′44″N, 82°52′29″W, collected under permit DRTO-2020-SCI-0015 to K.C.G.; Fig. S1). Across these three reef sites (Fig. S1), a total of 69 coral individuals (LKA = 10; LKB = 13; and DT = 46) from nine coral species were measured: *Agaricia agaricites* (*n* = 4), *C. natans* (*n* = 4), *M. cavernosa* (*n* = 10), *O. faveolata* (*n* = 4), *P. astreoides* (*n* = 15), *P. porites* (*n* = 2), *Pseudodiploria clivosa* (*n* = 6), *Pseudodiploria strigosa* (*n* = 12), and *S. siderea* (*n* = 12). Only visually healthy corals were sampled (average 5.4 on Coral Watch scale 1–6, Table S1). Corals were measured in waters at depths ranging from 1 to 3 m and with an average temperature of 27 °C at LK and 25 °C at DT.

#### In situ extracellular superoxide measurements associated with coral

To measure in situ extracellular superoxide concentrations, we used the recently developed DIver-operated Submersible Chemiluminescent sensOr (DISCO (29)) with the latest modifications (62). Briefly, DISCO is a hand-held submersible instrument that utilizes the chemiluminescent probe methyl Cypridina luciferin analog (MCLA, Santa Cruz Biotechnology), which has high specificity for superoxide (6, 49). When superoxide within the analyte fluid mixes with MCLA in the flow cell inside DISCO, photons are produced and measured (counts) in real time by an adjacent photomultiplier tube (PMT). Measured counts were converted to superoxide concentrations via daily calibrations using potassium superoxide (KO_2_) as detailed previously (29, 62). Superoxide dismutase (SOD), an enzyme that rapidly degrades superoxide (63), was periodically doped into the analyte to confirm the superoxide signal.

Here, DISCO was used on snorkel to collect in situ extracellular superoxide steady-state measurements associated with corals following the previous protocol (29). Briefly, measurements from individual coral were completed by obtaining a steady signal (∼30–120 s) from the following: (i) BGSW (SW >30 cm from corals at coral depth), (ii) coral water (<5 mm from coral surface without touching, moving steadily along surface to minimize BGSW entrainment), (iii) coral water dosed with SOD, and (iv) back to BGSW. All extracellular superoxide concentrations are presented as SW normalized, where the average counts associated with BGSW measured immediately prior to each coral sample is subtracted from the counts measured associated with each coral; the counts are then converted to superoxide concentrations using the site-specific calibration factor. Only visually healthy corals were sampled, and when possible, a visual assessment of coral health was assigned through photographs alongside the coral watch color chart (64).

#### Extracellular superoxide decay and production rates associated with coral

Immediately following in situ sampling for steady-state extracellular superoxide counts, 30 mL of water were collected at the surface of corals via a syringe for decay analysis. Syringes were stored at in situ temperatures in the dark briefly during transport from the dive site to a field laboratory, where they were analyzed following a previous protocol (46). In brief, known concentrations of a superoxide standard (average 37 nM) were spiked into unfiltered coral water and measured over time using DISCO. Decay rate constants were calculated using a pseudo-first-order decay equation (herein referred to as decay rates (22, 46)). Decay rate constants were also collected on-reef (>10 ft above all coral) and off-reef (∼100 m outside the reef). When available, extracellular superoxide production rates were calculated by multiplying respective decay rate constants by steady-state SW normalized extracellular superoxide concentrations (46).

### Coral larvae collection and analysis

#### Coral larvae collection and settlement

Approximately 200 *P. astreoides* coral larvae were collected in La Parguera, Puerto Rico, during spawning events in June 2017 to be measured for extracellular superoxide production (Department of Natural and Environmental Resources #2016-IC-187 to L.M.R.). Larvae were transported back to the Marine Biological Laboratory (MBL) in containers with SW. They were kept alive and healthy in clean beakers (Fig. S2a) with 0.22 µM filtered Martha's Vineyard Sound SW (MVSSW) that was temperature controlled at 26 °C. Beakers were covered with a transparent film and housed within a temperature (26 °C)—and light-controlled (250 µmol photons m^−2^ s^−1^, 12:12 h light:dark cycle) flow-through tray at the MBL. SW and glass beakers were changed daily to prevent settling.

After 2 weeks, settling was encouraged for ∼44 swimming larvae by using a transfer pipette to gently place larvae onto four conditioned ceramic tiles (Fig. S2b). Larval settlement was visually confirmed using a microscope (Fig. S2c and d) and budding development was analyzed each sampling day to account for multiple polyps during measurements (Fig. S2e). The four tiles were kept inside two covered plastic aquaria containing 0.22 µM filtered MVSSW. These were housed within the same flow-through system as the larvae with water changes three to four times a week. For the first 90 days, polyps were fed with New Life Spectrum Nutri/Cell Coral Food Filter Feeder Microcapsules Pet Food. After 90 days, the polyps were fed three times a week with live *Artemia*.

#### Superoxide chemiluminescent measurements using the FeLume

For laboratory analysis, superoxide was measured with a benchtop flow injection FeLume Mini system (Waterville Analytical, Waterville, ME, USA (65)). The FeLume utilizes the same chemical methods as DISCO yet is a benchtop instrument with external peristaltic pumps and a PC computer that is used for operation and data collection. The FeLume was used according to the protocol described in prior studies (6, 28, 30, 49, 66).

#### Extracellular superoxide measurements of aquaria-hosted *P. astreoides* swimming larvae and settled polyps

*Porites astreoides* swimming larvae were measured using the FeLume according to procedures used for bacteria and coral larvae as described in previous studies (3, 28). All measurements used artificial SW amended with 75 µM DTPA (aged artificial SW; AASW) for the analyte medium. In brief, a sterile 0.22 µM Millipore syringe filter was placed in the FeLume analyte line. AASW was pumped across it at a flow rate of 2.25 mL/min until a steady signal was obtained (30 s to 2 min) for BGSW. A larvae-loaded filter was placed into the analyte line with the pump stopped. When the pump was restarted, the extracellular superoxide associated with larvae was carried by the AASW and mixed with the MCLA to be measured as a chemiluminescent signal by the PMT. Superoxide signal from each sample was measured until a steady signal was obtained (∼30 s to 2 min). The intake was then placed in a beaker with AASW amended with SOD (0.8 U mL^−1^, final). Larva(e) densities (1, 5, 10, and 20) were measured in triplicate (Fig. S4).

*Porites astreoides* settled polyps were measured using the FeLume by placing the analyte intake into a beaker containing AASW and a ceramic tile with settled polyps, which was acclimated to AASW for ∼10 min prior to measurements. To complete a measurement, a steady signal was collected by sequentially using the intake to measure the following: (i) AASW only in a beaker, (ii) AASW with a tile in a beaker to measure BGSW (∼3 cm away from the tile), (iii) AASW near the surface of a coral polyp (∼1 mm away from the polyp), (iv) AASW with a tile in a beaker to measure BGSW (∼3 cm away from the tile), and (v) AASW near the surface of another coral polyp (∼1 mm away from the polyp). This pattern was repeated for each coral polyp on one tile face, with BGSW measurements between each polyp measurement. The tile itself would then be analyzed by moving the intake slowly along the surface of the tile, avoiding any polyps. After all polyps on one side of the tile were measured, the intake was placed into a Falcon tube with AASW amended with SOD (0.8 U mL^−1^, final) to confirm superoxide signal measurements. This process was completed for each side of all four tiles on each sample day.

#### Aquaria-hosted adult *P. astreoides* superoxide measurements

Extracellular superoxide concentrations associated with four adult *P. astreoides* corals hosted in two aquaria at the MBL were measured. Measurements were made with the FeLume and were measured using similar techniques to the in situ superoxide measurements, yet the SOD was doped into a beaker of coral water rather than inline.

#### Calibrations for superoxide concentration

The measured superoxide signal from the FeLume was converted to superoxide concentrations following a similar protocol as described above for DISCO and as described in depth from previous studies (3, 28, 30). The site-specific calibration factor that was used to calculate the in situ superoxide concentrations was 0.0144 and 0.0197 pM Lum^−1^ at Looe Key and Dry Tortugas, respectively.

### NOX-like genes in adult scleractinian corals

To search for NOX-like genes, we queried cnidarian NOX protein sequences identified by Taenzer et al. (38) against amino acid sequences from publicly available annotations of scleractinian genome and transcriptome assemblies using blastp (BLAST). Significant matches were defined as a complete sequence *E*-value <1e−20 and score >200.

The screened genome and transcriptome assemblies were selected to maximize the number of families represented and capture the phylogenetic diversity of Scleractinia. Only annotated genomes and transcriptomes were considered. We included one genome assembly per family, preferring genome assemblies from species or genera measured with DISCO. This selection process resulted in nine genomes and one transcriptome, representing five families from each of the “robust” and “complex” clades (Table S2).

Amino acid sequence alignments and annotations were visually inspected in Geneious. Sequences that contained ferric reductase, FAD-binding eight domains, and NAD-binding six domains were identified as putative NOX-like genes.

Putative NOX-like genes were further classified by (i) sequence alignment incorporating reference eukaryotic amino acid sequences used by Taenzer et al. (38) using Clustal Omega (67) and (ii) phylogenetic analyses using maximum likelihood in RAxML v.8.2.11 (68) (GAMMA BLOSUM62 protein model; rapid hill climbing). Statistical support for inferred clades was assessed through bootstrapping (200 replicates).

## Supplementary Material

pgag075_Supplementary_Data

## Data Availability

All data are included in the manuscript and Supplementary material.
