# Supplementary material for: Extracellular superoxide production by Porites species provides insight into controls on coral physiology
Source: PNAS Nexus. 2026 Mar 19;5(4):pgag075. doi: 10.1093/pnasnexus/pgag075 (PMC13069887; doi:10.1093/pnasnexus/pgag075)
Supplement: pgag075_Supplementary_Data [file pgag075_supplementary_data.zip › SupportingInformation.docx]

**Supporting Information for**

**Crystallization of stardust analogs under an electron flux atmosphere**

**Rakibul A. Shohan^1^, Cody Cly^1^, Angela Speck^1^, Benjamin Sargent^2,3^ Joseph A. Nuth III^4^, Alan Whittington^5^, Arturo Ponce^1*^**

Arturo Ponce Ph.D.

Email: arturo.ponce@utsa.edu

**This PDF file includes:**

Supporting text

Figures S1 to S7

Tables S1 to S2

Legends for Datasets S1 to S7

**Other supplementary materials for this manuscript include the following:**

Datasets S1 to S7

Supporting Information Text

**11-12μm features**

The 11-12μm feature matches lab spectra of glassy/amorphous alumina, not corundum, which has a narrower feature around 13 μm (*1-4*). The presolar grains that have been studied are a mix of α- and amorphous (ref. 25 from the main manuscript), but apparently one grain has been studied that may be η- or γ-alumina. We also need to recognize that while presolar grains are a guide – they have traveled through more energetic environments en route to the forming solar system and some may have been crystalized but not destroyed, thus preserved their isotopic presolar signature, but not their nascent crystal structure.

**Morphology and EDS**

Scanning electron microscopy (SEM) analysis of as-synthesized alumina smokes revealed agglomerated particles with a variety of sizes in the micron range. The micrograph shows that the particle morphology was predominantly equant. To complement the SEM analysis and investigate the chemical composition of the samples, energy-dispersive X-ray spectroscopy (EDS) was performed simultaneously. The EDS spectrum obtained from as-synthesized alumina smokes confirmed the presence of aluminum and oxygen in similar intensity proportions.

**Grain sizes with annealing**

Through thermal treatment, as-synthesized amorphous alumina smoke particles undergo microstructural evolution. Initial annealing at 1000˚C induced crystallization of η-Al_2_O_3_ with a grain size of ~15 nm. Subsequent annealing at 1300˚C facilitates a phase transformation to the thermodynamically stable α-Al_2_O_3_ polymorph, and grain size increasing to ~300 nm.

**PDFgui fitting**

Simulated η-Al_2_O_3_ phase was fitted to experimentally obtained ePDF G(*r*) data from amorphous alumina smokes in the short-range order of 1.5 - 8Å. Lattice parameters (a, b, c) and atomic displacement parameters (ADPs) were refined to achieve a better fit. The scale factor and Q_damp_ were also fitted at 1.3 and 0.08, respectively. The angle of the unit cell was not considered during fitting.

**Transmission electron microscope parameters**

The images and electron diffraction patterns of the annealed samples were recorded in a TVIPS 16-mega pixel F416 CMOS camera attached to a JEOL 2010F microscope operated at 200 kV. The electron diffraction patterns of the electron beam irradiation were collected in a Rio Camera CMOS adapted to a JEOL cold-FEG JEM-F200 microscope operated at 80 kV and 200 kV. The experiments performed at low temperature were collected in a CCD camera attached to a JEOL ARM200F microscope operated at 200 kV.

**ePDF data process**

The electron flux was calculated by measuring the current density (ρ) on registered in the fluorescent screen of the microscope by using the following formula:

$\sigma=\rho\times{(0.8M)}^{2}$ [1]

where σ is the electron flux (number of electrons per area per second, $\bar{e}{/m}^{2}/s$), ρ is the current density measured on the screen viewing (pA/cm^2^), *M* is the magnification on the screen (5).

Electron diffraction patterns were processed as follows:

1. From the electron diffraction pattern, we obtain the radial distribution intensity and subsequently normalized using form factors to obtain the structure function S(Q).
2. Then, the sine Fourier transform is obtained from the structure function to obtain the electron pair distribution function (ePDF) G(r).
3. The ePDF of all samples are processed using RDFTools and eRDF software packages (*6, 7*). For electrons the parameterization of atomic scattering factors used in the eRDF software is reported by Kirkland and Labato (*7, 8*).

**Electron Flux Calculation**

The electron flux is a measure of the number of electrons passing through a unit area per unit time. Electron flux *Γ_z_* (in the z direction) can be calculated using the electron density (*n_e_*) and electron temperature (*T_e_*), along with knowledge about electron velocity distribution, typically assumed to follow the Maxwellian distribution in a plasma context (*9-12*). The average velocity of electrons ν̅ for a Maxwellian distribution is given by

$\bar{v}=\sqrt{\frac{8k_{B}T_{e}}{\pi m_{e}}}$ [2]

Where:

ν̅ is the thermal velocity of electrons (m/s), *k_B_* is the Boltzmann constant (1.38 x 10^-23^ J/K), *T_e_* is the electron temperature (K), and *m_e_* is the mass of an electron (9.11x 10^-31^ kg). Electron flux *Γ_z_* is related to *n_e_* and ν̅ by:

$\Gamma_{z}=\frac{n_{e}\bar{v}}{4}$ [3]

$\Gamma_{z}=\frac{1}{4}n_{e}\sqrt{\frac{8k_{B}T_{e}}{\pi m_{e}}}$ [4]

The average energy per electron in the z direction, E_ave,z_ is given by:

$E_{ave,z}=\frac{k_{B}T_{e}}{2}$ [5]

For planetary nebulae, a typical value of *T_e_* = 10,000 K and *n_e_* ≈10^4^ electrons/cm^3^. The calculated electron flux *Γ_z_* is ~10^15^ e^–^/m^2^/s from Eq. 4, and calculated average kinetic energy E_ave,z_ ≈ 1 eV from Eq. 5.

Planetary nebulae can produce a substantial population of suprathermal electrons following a κ-distribution (*13-14*). In the case of needing electrons with higher energies to overcome a crystallization threshold, we can use equation (1) from (13) to calculate both the *n_e_* and ν̅ while setting a lower limit to the electron energy:

$n\left( v \right)dv=\frac{4N}{\sqrt{\pi}w_{0}^{3}}\left( \frac{\Gamma\left( \kappa+1 \right)}{\kappa^{3/2}\Gamma\left( \kappa-\frac{1}{2} \right)} \right)\frac{v^{2}}{\left( 1+{v^{2}}/\left[ \left( \kappa-\frac{3}{2} \right)w_{0}^{2} \right] \right)^{\kappa+1}}dv$ [6]

where *n*(*v*) is the number of electrons with speeds between *v* and *v* + d*v*, w_0_ is the velocity, and the value of kappa is chosen as 20 based on (*13, 14*). The lower limit of 1 keV is chosen as a demonstration. While most of the electrons are below 1 keV, suprathermal distributions yield a much higher number of electrons >1 keV than would be the case for a Maxwellian/thermal distribution. The calculated electron flux *Γ_z_* (>1 keV) ≈ 10^9^ e^–^/m^2^/s.


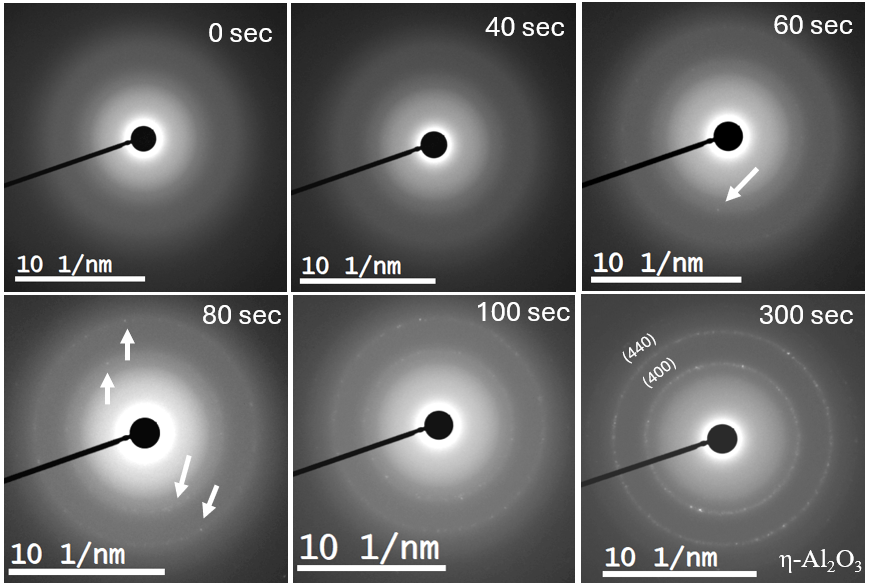


Fig. S1. In-situ observation of SAED patterns collected from nearly identical regions as a function of electron exposure duration at electron flux of 10^22^ electrons/m^2^/s under 80 keV electron energy, corresponding to a current density registered in the microscope of 86 pA/cm^2^. Only the amorphous phase is present at 0 sec and 40 sec. Crystallization starts at around 60 seconds of irradiation, and gradual developments of Debye-Scherrer rings after 80, 100 and 300 seconds of irradiation correspond to (400) and (440) planes of η-Al_2_O_3_. White arrows indicate initial spots of crystallinity.


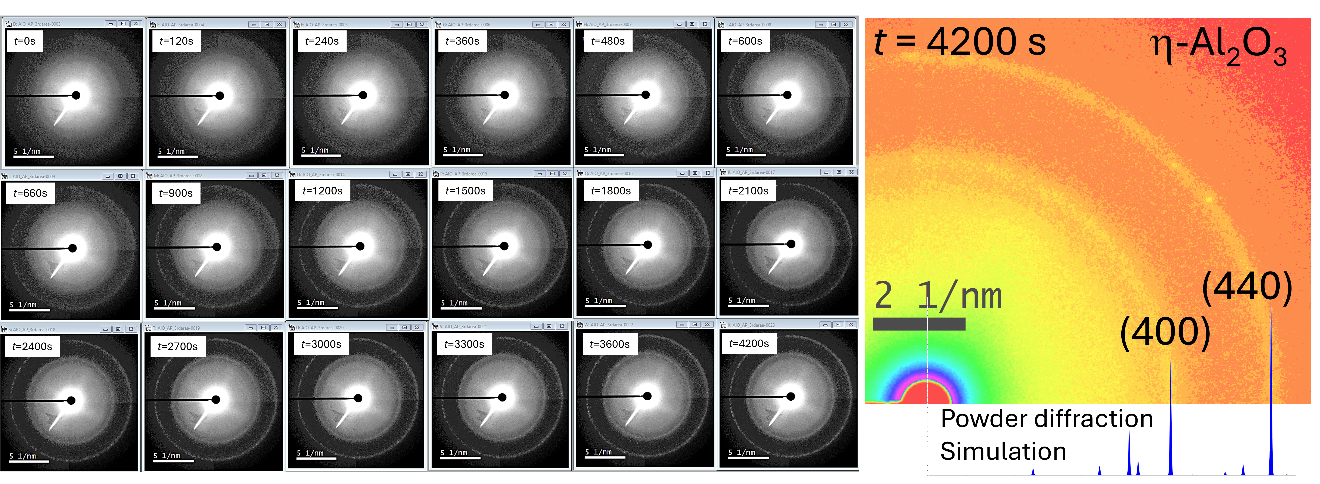


Figure S2. Sequence of selected area electron diffraction patterns collected at a reduced electron flux of 10^21^ electrons/m^2^/s under 200 keV electron energy, corresponding to a current density registered in the microscope of 8.8 pA/cm^2^. The color SAED pattern corresponds to the time 4200 seconds and is indexed to show the family of planes of the η-Al_2_O_3_ phase.


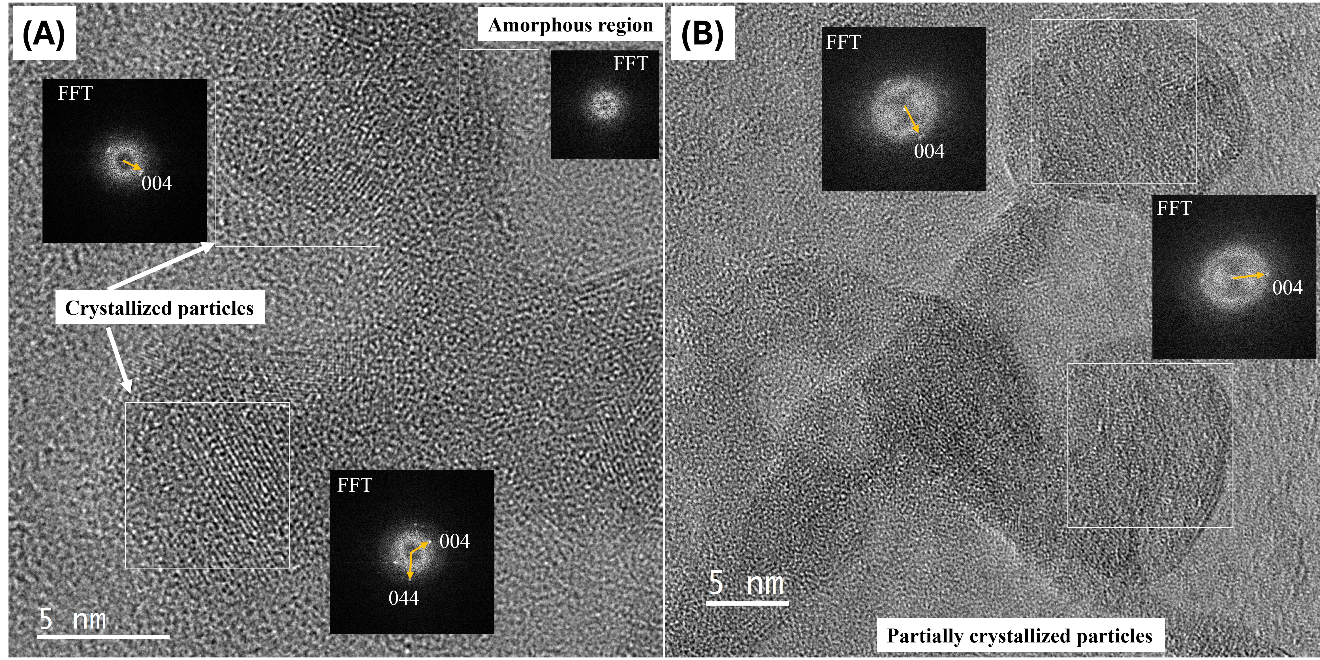


**Figure S3.** HRTEM micrographs of irradiated regions exposed at 4200 seconds using an electron flux of (10^21^ $\bar{e}{/m}^{2}/s$), which corresponds to a comulative electron dose of 42x10^24^ $\bar{e}{/m}^{2}$, (A) crystallized particles and amorphous layer, (B) partially crystallized surrounded by amorphous regions.
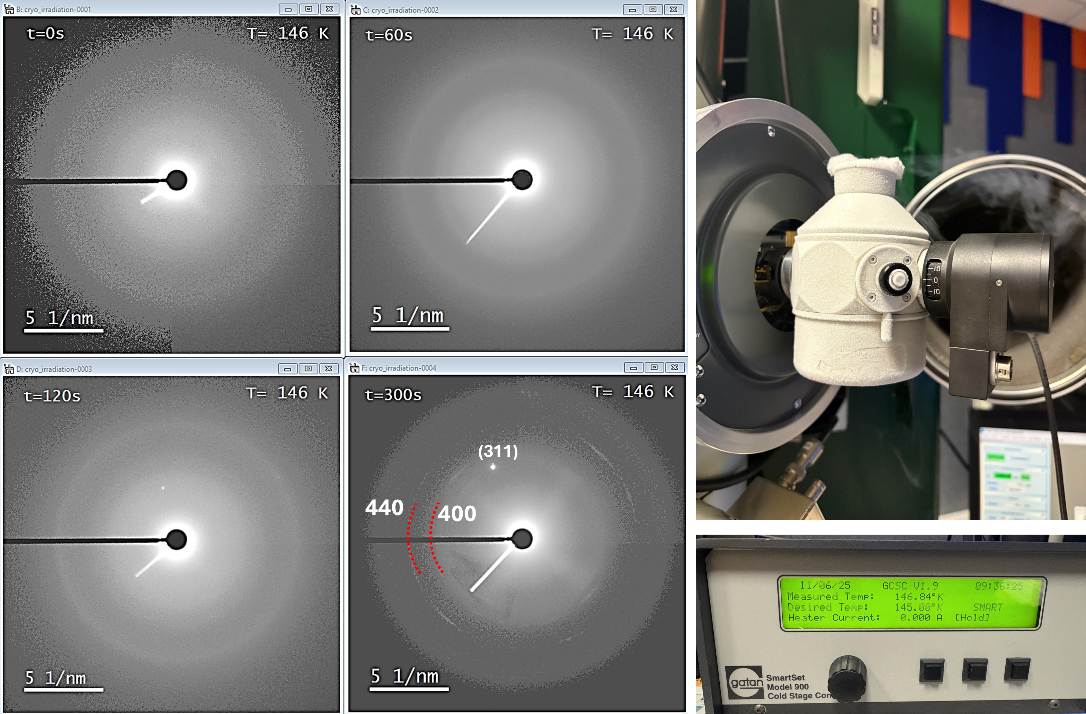


**Figure S4.** Left: Sequence of SAED patterns collected during the in-situ electron beam irradiation at 86 pA/cm^2^ (10^22^ $\bar{e}{/m}^{2}/s$). The irradiation times are labeled in the SAED patterns acquired at 200 keV and at low temperature (146 K). Debye-Scherrer rings correspond to (400) and (440) planes of η-Al_2_O_3_ phase. Right: experimental setup of the TEM liquid nitrogen holder and cold stage controller. Formation of diffraction rings are observed after 300 seconds of irradiation.


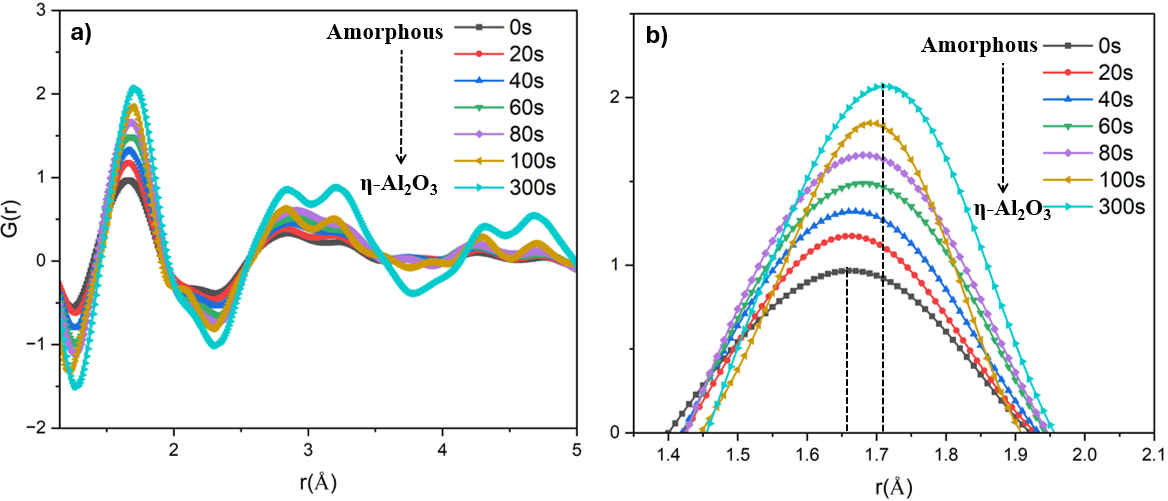


Fig. S5. (a) Evolution in pair distribution functions revealed through the analysis of SAED patterns collected from consistent specimen regions during in-situ electron irradiation at 80 keV. (b) Progression of the first peak radial shifts as a function of irradiation time.


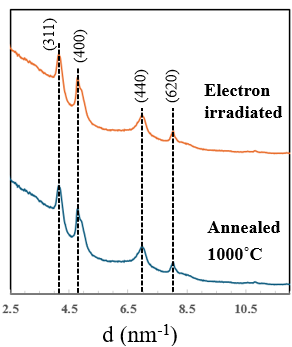


Fig. S6. Intensity profiles measured from SAED patterns collected for alumina smokes both in annealing and electron irradiation experiments.


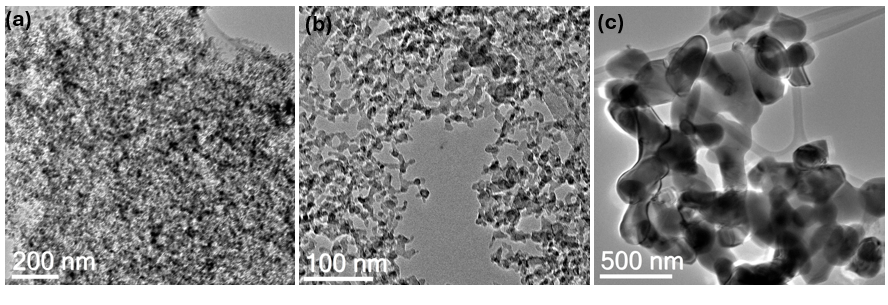


Fig. S7. (a) Coalescence of alumina smokes particles in amorphous state, (b) formation of nanoscale crystalline η-Al_2_O_3_ grains after thermal annealing at 1000˚C, and (c) increased crystalline size and transformation to α-Al_2_O_3_ observed after annealing at 1300˚C.

Table S1. Parameters of electron exposure in in-situ SAED experiments.

| Flux  e^-^/ m^2^/s | Voltage (keV) | Magnification/field of view (FOV) | Current density (ρ) pA/cm^2^ | η-Al_2_O_3_ | |
| --- | --- | --- | --- | --- | --- |
|  |  |  |  | First spot (Sec) | Polycrystal (Sec) |
| 10^21^ | 200 | 200,000/16,129 nm^2^ | 8.8 | 480 | 4200 |
| 10^22^ | 80 | 200,000/16,129 nm^2^ | 86 | 60 | 300 |
| 10^22^ | 200 | 200,000/16,129 nm^2^ | 86 | 60 | 300 |
| 10^24^ | 200 | 800,000/1,560 nm^2^ | 165 | -- | 300, 360 & 720 |

Table S2. Optimized fitting parameters for PDFgui.

| Parameters | η-Al_2_O_3_ | Fitted η-Al_2_O_3_ |
| --- | --- | --- |
| a | 3.95 | 3.68 |
| b | 3.95 | 3.68 |
| c | 3.95 | 3.68 |
| ADPs | NA | 0.01 |

**SI References**

1. B. Begemann et al, Aluminum oxide and the opacity of oxygen-rich circumstellar dust in the 12-17 micron range. *The Astrophysical Journal* **476(1)**, 199 (1997).
2. T. Eriksson, A. Hjortsberg, G. Niklasson, C.G. Granqvist, Infrared optical properties of evaporated alumina films. *Applied Optics* **20(15)**, 2742-2746 (1981).
3. F. Gervais (1991). Chapter 14: Corundum (Al2O3). In E. D. Palik (Ed.), Handbook of Optical Constants of Solids, Volume 2 (pp. 371-396). Academic Press.
4. M. E. Thomas, S.K. Andersson, R.M. Sova, R. I. Joseph, Frequency and temperature dependence of the refractive index of sapphire. *Infrared physics & technology* **39(4)**, 235-249 (1998).
5. Ortega et al. Structural damage reduction in protected gold clusters by electron diffraction method, *Adv Struct Chem Imag* **2**, 12 (2016).
6. D. R. G. Mitchell, T. C. Petersen, RDFTools: A software tool for quantifying short-range ordering in amorphous materials, *Microscopy Research & Technique* **75**, 153-163 (2012).
7. J. Shanmugam, K.B. Borisenko, Y.J. Chou, A.I. Kirkland, eRDF Analyser: An interactive GUI for electron reduced density function analysis. *SoftwareX* **6**, 185-192 (2017).
8. I. Lobato, D. Van Dyck, An accurate parameterization for scattering factors, electron densities and electrostatic potentials for neutral atoms that obey all physical constraints. *Acta Cryst.* **70**, 636-649 (2014).
9. D. E. Osterbrock, Electron Densities in Planetary Nebulae. *Astrophysical Journal* **131**, 541 (1960).
10. G. S. Khromov, Evolution of planetary nebulae and their nuclei-the density of planetary nebulae and its evolutionary changes. *Soviet Astronomy* **20**, 543 (1976).
11. L. Stanghellini, J. B. Kaler, Electron densities in planetary nebulae. *Astrophysical Journal* **343**, 811-827 (1989).
12. Y. Zhang et al, Electron temperatures and densities of planetary nebulae determined from the nebular hydrogen recombination spectrum and temperature and density variations. *Monthly Notices of the Royal Astronomical Society* **351(3)**, 935–955 (2004).
13. D. C. Nicholls, M. A. Dopita, R. S. Sutherland, Resolving the electron temperature discrepancies in H II regions and planetary nebulae: κ-distributed electrons. *Astrophysical Journal* **752(2)**, 148 (2012).
14. Y. Zhang, B. Zhang, X. W. Liu, On the nonthermal κ-distributed electrons in planetary nebulae and h ii regions: The κ index and its correlations with other nebular properties. *Astrophysical Journal* **817(1)**, 68 (2016).
15. L. Smrčok, V. Langer, M. Halvarsson, S. Ruppi, A new Rietveld refinement of κ-Al_2_O_3_. *Zeitschrift fur Kristallographie* **216(7-2001)**, 409–412 (2001).
16. E. Husson, Y. Repelin, Structural studies of transition aluminas. Theta alumina. *European Journal of Solid State and Inorganic Chemistry* **33**, 1223–1231 (1996).
17. E. N. Maslen, V. A. Streltsov, N. R. Streltsova, N. Ishizawa, Y. Satow, Synchrotron X-ray study of the electron density in α-Al_2_O_3_. *Structural Science* **49(6)**, 973–980 (1993).
18. H. Saalfeld, M. Wedde, Refinement of the crystal structure of gibbsite, A1(OH)_3_. *Zeitschrift für Kristallographie-Crystalline Materials* **139(1-6)**, 129–135 (1974).
19. Y. Repelin, E. Husson, Etudes structurales d'alumines de transition. I-alumines gamma et delta. *Materials Research Bulletin* **25(5)**, 611–621 (1990).

**Dataset S1.** Pair distribution functions measured in in-situ electron irradiation at 200 keV.

**Dataset S2.** X-ray diffraction of alumina smokes annealed at 1000C and at 1300C.

**Dataset S3.** Pair distribution functions measured for ex-situ thermal annealing and compared with simulated different phase of alumina.

**Dataset S4.** Fitting of simulated η-Al_2_O_3_ phase to experimental G(r) data for amorphous alumina in the short-range.

**Dataset S5.** Pair distribution functions measured in in-situ electron irradiation at 80 keV.

**Dataset S6.** Intensity profiles from SAED patterns for alumina smokes both in annealing and electron irradiation experiments.

**Dataset S7.** Python script for electron flux estimation (>1keV) for planetary nebulae.
