# Supplementary material for: Extracellular superoxide production by Porites species provides insight into controls on coral physiology
Source: PNAS Nexus. 2026 Mar 19;5(4):pgag075. doi: 10.1093/pnasnexus/pgag075 (PMC13069887; doi:10.1093/pnasnexus/pgag075)
Supplement: pgag075_Supplementary_Data [file pgag075_supplementary_data.zip › SupportingInformation_TableS1.docx]

| Flux  e^-^/ m^2^/s | Voltage (keV) | Magnification/field of view (FOV) | Current density (ρ) pA/cm^2^ | η-Al_2_O_3_ | |
| --- | --- | --- | --- | --- | --- |
|  |  |  |  | First spot (Sec) | Polycrystal (Sec) |
| 10^21^ | 200 | 200,000/16,129 nm^2^ | 8.8 | 480 | 4200 |
| 10^22^ | 80 | 200,000/16,129 nm^2^ | 86 | 60 | 300 |
| 10^22^ | 200 | 200,000/16,129 nm^2^ | 86 | 60 | 300 |
| 10^24^ | 200 | 800,000/1,560 nm^2^ | 165 | -- | 300, 360 & 720 |
