# Supplementary material for: Extracellular superoxide production by Porites species provides insight into controls on coral physiology
Source: PNAS Nexus. 2026 Mar 19;5(4):pgag075. doi: 10.1093/pnasnexus/pgag075 (PMC13069887; doi:10.1093/pnasnexus/pgag075)
Supplement: pgag075_Supplementary_Data [file pgag075_supplementary_data.zip › SupportingInformation_TableS2.docx]

| Parameters | η-Al_2_O_3_ | Fitted η-Al_2_O_3_ |
| --- | --- | --- |
| a | 3.95 | 3.68 |
| b | 3.95 | 3.68 |
| c | 3.95 | 3.68 |
| ADPs | NA | 0.01 |
